# Allelopathic effects of bioactive aqueous extracts on the growth and development of *Solanum lycopersicum* L

**DOI:** 10.3389/fpls.2025.1536309

**Published:** 2025-07-28

**Authors:** Tetiana Solodka, Olesia Solodka

**Affiliations:** ^1^ Educational and Scientific Institute of Agroecology and Land Management, National University of Water and Environmental Engineering, Rivne, Ukraine; ^2^ Sections of Biology and Agricultural Sciences, Rivne Regional Branch of the Ukrainian Junior Academy of Sciences, Rivne, Ukraine

**Keywords:** allelopathic plant extracts, tomato growth regulation, phytotoxic effects, natural biostimulants, seed germination dynamics

## Abstract

This study examines the allelopathic effects of aqueous seed extracts from *Brassica napus* L., *Mentha piperita* L., *Passiflora incarnata* L., and *Sinapis alba* L. on the growth and development of *Solanum lycopersicum* L. The application of the extracts influenced seed germination dynamics and altered the accumulation of photosynthetic pigments. The extract of *Sinapis alba* L. led to the highest increase in chlorophyll a and b levels, while *Brassica napus* L. showed the strongest stimulation of seed germination. One-way ANOVA confirmed statistically significant differences between treatments. The results support the potential of selected botanical extracts as natural biostimulants. Further investigation is required to assess their efficiency in field conditions and their interaction with soil microbiota.

## 1 Introduction

Intensive research on volatile substances such as ‘apple air’ or ethylene gas in the 1920s and 30s eventually identified ethylene (Molisch, 1937), as well as allelopathic compounds such as allelochemicals (Kholodny, 1957), leading to the formulation of the concept of allelopathy. The work of the famous Viennese physiologist G. Molisch was invaluable in deepening the understanding of chemical interactions in plants; he also coined the term ‘allelopathy’. Molisch understood allelopathy broadly, referring to the influence of one plant on another (Shirgapure and Ghosh, 2020). Significant progress in understanding plant interactions occurred in the late 18th and early 19th centuries with the humus theory of plant nutrition. Antoine de Candol’s idea of chemical interactions between plants, expressed in his Studies on Plant Physiology and his crop rotation theory, argued that under natural conditions there is no monoculture and plants alternate in the same area. De Candol saw the need for such a crop rotation, as each plant accumulates root secretions that are harmful to itself but necessary for the existence of other plants (Hierro and Callaway, 2021). Allelopathy, as a cycle of physiologically active substances within the cenosis, is crucial for farming systems. Excess of physiologically active substances in the cenosis environment has a harmful effect on plant growth, as well as their deficiency (Chou and Wang, 2023). Two thousand three hundred years ago, the ‘father of botany’ Theophrastus wrote in his Studies on Plants: ‘Sometimes one tree harms another, taking away its food and interfering with its way of life. Bad neighborhood with ivy, bad with alfalfa: they, so to speak, destroy all trees, as sorrel turned out to be: it destroys even alfalfa’ (Khamare et al., 2022). The problem of allelopathy was significantly developed in the works of German scientists. Among the first studies was that of G. R. Bode, who investigated the effect of wormwood volatile emissions on fennel and the allelopathic properties of walnut and black walnut. After this study, the allelopathic properties and biochemistry of wormwood emissions were studied by H. Schwer (Grodzinsky, 1997). Separate studies of the role of chemical interactions in natural phytocoenosis are of interest in the works of G.F. Linskens and R. Klapp on the secretion of sugars and amino acids by roots and leaves, the effect of various secretions and extracts on plant growth (Hussain et al., 2021). The work of V. Fleig and his colleagues on the decomposition of organic matter in the soil and the formation of physiologically active humus-like substances is also important for allelopathy (Krupa and Figurskaya, 1990). Significant progress in allelopathic research has been observed in recent years in the United States. K.H. Mueller at the University of California is studying the mechanisms of allelopathic effects of American wormwood, California wormwood and black mustard on dry prairie vegetation, as well as the effects of eucalyptus and shrubs on annual grasses in wetter mountain conditions. E.L. Rice of the University of Oklahoma is investigating the role of allelopathic interactions between prairie plants and nitrogen-fixing microorganisms in a sequential series of successions during the revegetation of abandoned cropland. F. Woods and J. McCormick study the interaction of forest species, and T.M. McCall studies physiologically active substances formed during the microbiological decomposition of post-harvest residues (Li et al., 2019; Kalisz et al., 2021). The system of methods for studying allelopathic interactions between plants has not yet been sufficiently developed. Most of the existing methods are more or less successful adaptations for the needs of allelopathy, which, of course, do not meet the requirements of in-depth research. Therefore, the development of specific allelopathic methods is constantly needed (Latif et al., 2017). Our research focuses on the study of the allelopathic activity of *Brassica napus L., Mentha piperita L., Passiflora incarata L., Sinapis alba L.* through their living exudates. While *Brassica napus L., Mentha piperita L., Passiflora incarata L., Sinapis alba L.* contain bioactive compounds with potential regulatory effects on plant growth, their natural coexistence with tomatoes in agricultural conditions is not well documented. In this regard, the question arises whether these plants significantly affect the growth of tomatoes in natural conditions. The choice of these particular plants was based on their known biochemical properties and allelopathic effects. *Brassica napus L.* has been known for over four thousand years. Some researchers consider it to be native to Europe, in particular the northwestern coastal areas (coastal lands of Sweden, the Netherlands and the UK), while others point to the Mediterranean. The fact that rapeseed cultivation has been widespread in Asia, especially in India, supports the latter theory (Hierro and Callaway, 2021). The allelopathic potential of *Brassicaceae* crops (in particular, *Brassica napus L.* and *Sinapis alba L.*) is confirmed by the content of glucosinolates, which are converted into several isothiocyanates by enzymatic (myrosinase) activity and have allelopathic activity (Jabran, 2017). Recent studies have explored the specificity of isothiocyanate action on cellular targets, highlighting their role in disrupting auxin transport, modifying membrane permeability, and inducing oxidative stress in target plants (Zhang et al., 2020; Kostina-Bednarz et al., 2023). Moreover, the use of solvents such as DMSO to enhance the bioavailability of these compounds has been discussed as a methodological advance in allelopathic research (Li et al., 2019). *Brassica napus L.* has allelopathic properties due to the presence of glucosinolates, which are decomposed into isothiocyanates, phytotoxic compounds, under the influence of the myrosinase enzyme. They effectively inhibit the growth of parasitic and non-parasitic weeds, as shown by the results of the study of the effect of B. napus on Orobanche crenata, a parasitic weed on Pisum sativum (Ahmed et al., 2024). *Mentha piperita L.* is widely known for its medicinal properties, primarily for its ability to soothe gastrointestinal discomfort and its use in aromatherapy to relieve stress. It has been cultivated for centuries for both culinary and medicinal purposes. Peppermint oil, extracted from its leaves, is rich in menthol, which has various therapeutic uses. The allelopathic properties of the plant can also affect the growth of neighboring plants by releasing chemical compounds into the soil (Chalkos et al., 2010). *Mentha piperita* produces essential oils that can act as growth inhibitors. The interest in this plant in the study was aroused by the article by Argyropoulos et al (Liu et al., 2013), who reported a negative effect on seed germination and seedling length for *Mentha* sp*icata* essential oils not only in the case of weeds, but also for tomato and cotton crops, while similar results were obtained for aqueous extracts of *Mentha piperita* on tomato seed germination and seedling growth. On the contrary, (Chalkos et al., 2010). reported that the addition of *Mentha* sp*icata* composts to growth media with different rates significantly enhanced tomato growth while suppressing weeds. *Passiflora incarnata* contains alkaloids with potential allelopathic effects. According to a study (Khanh et al., 2005), the interest in studying the allelopathic effects of *Passiflora incarnata* arises from its excellent response to weed growth. Despite the fact that most of the plants studied showed strong allelopathic activity, *Passiflora incarnata* did not significantly inhibit the spontaneous growth of rice weeds under greenhouse conditions at a dose of 1 t/ha. This discovery points to potentially unique mechanisms of allelopathy that require further study to find out the reasons for its less pronounced effect compared to other plants. These species were chosen not only because of their well-documented allelopathic activity, but also because they are commonly found in agricultural and natural ecosystems where tomatoes are cultivated. Their potential interaction with *Solanum lycopersicum L.* in the field, as companion plants, rotational crops or natural vegetation, makes them suitable subjects for research. In addition, the study of the allelopathic effects of non-weed species on agricultural plants provides insight into possible applications for sustainable agriculture, such as natural growth regulators or bioherbicides. Although the allelopathic effects of weeds on crops is an important area of research, this study specifically focuses on these selected species because of their potential use in organic farming practices. This study fills a gap by assessing the impact of plant-derived compounds on tomato cultivation, contributing to a broader understanding of allelopathic interactions in agroecosystems.

## 2 Addressing common challenges in the use of protein-lipid complexes for the cultivation of *Solanum lycopersicum* L.

The allelopathic properties of plants are determined by the quantitative and qualitative composition of exudates that enter the environment as a result of plant life processes and after its decay. These substances can accumulate in the soil and affect the growth of other species (Xu et al., 2023). We propose to use bioactive aqueous extracts from *Brassica napus L., Mentha piperita L., Passiflora incarnata L., Sinapis alba L.* as physiologically active substances for growing agricultural plants, in particular tomatoes (*Solanum lycopersicum L.*). These bioactive substances can regulate plant development and influence the growth processes of crops. The more traditional approach in allelopathic research often focuses on the interaction between weeds and cultivated crops, as weeds usually coexist with and compete with crops in natural and managed ecosystems. The study aims to assess whether the selected species can be used as natural growth regulators, providing an alternative approach to chemical treatments in tomato cultivation. The study of bioactive aqueous extracts derived from these plants contributes to a broader understanding of allelopathic interactions in agroecosystems. To achieve this, we set the following goals: First, we evaluated the strength of seed germination under certain treatment conditions in petri dishes. Secondly, we evaluated the germination of tomato seeds under certain treatment conditions in sand. Third, we analysed the content of photosynthetic pigment in *Solanum lycopersicum L.* plants treated with botanical extracts. Finally, we have substantiated the possibility of using bioactive aqueous extracts of *Brassica napus L., Mentha piperita L., Passiflora incarnata L., Sinapis alba L.* as physiologically active substances for growing tomatoes from seeds. The aim of the study was to investigate changes in tomato germination processes under the influence of these bioactive aqueous extracts.

**Figure 1:**
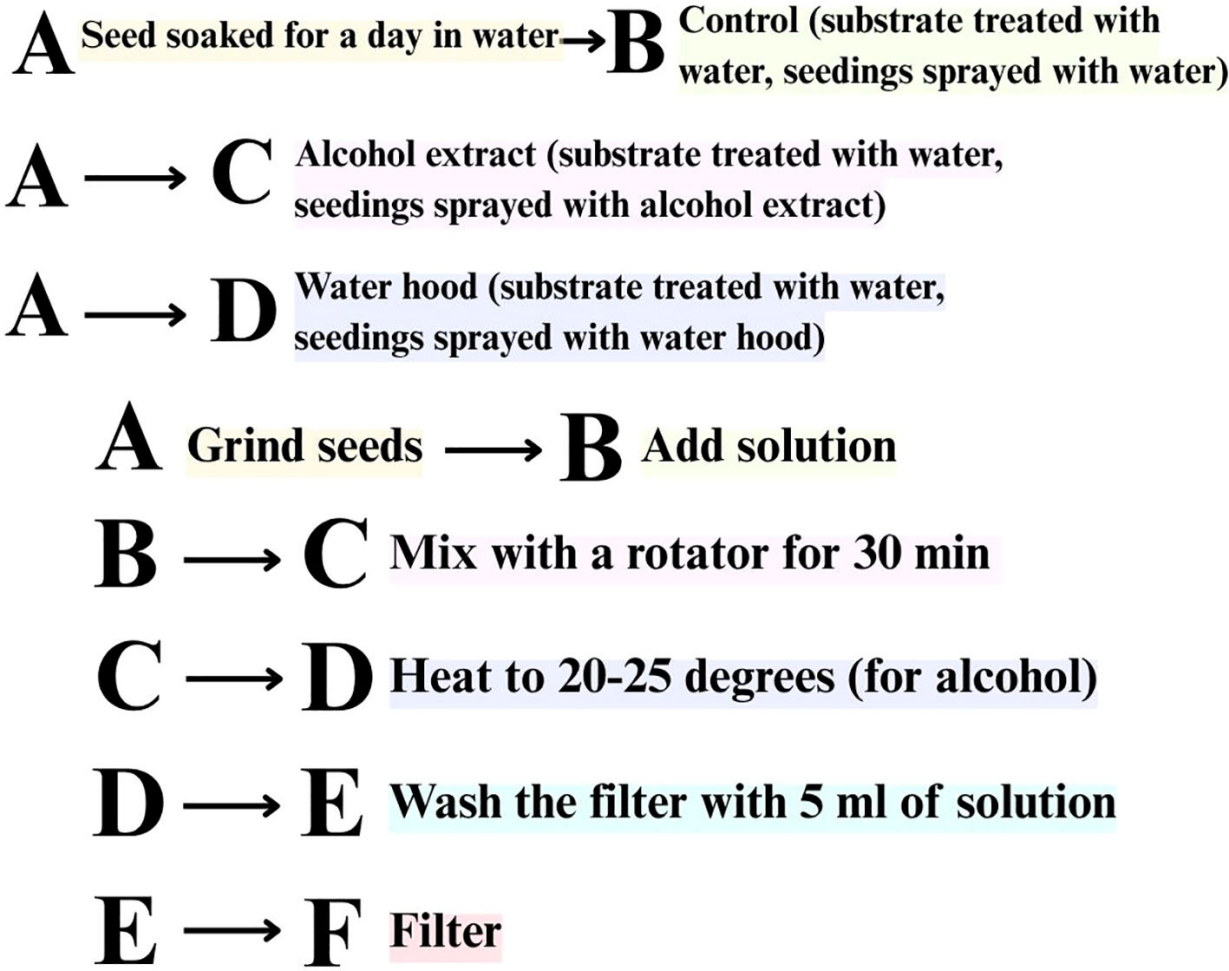
Scheme of preparation and application of botanical extracts used in the treatment of Solanum lycopersicum L. plants. The upper part illustrates the treatment variants (control, alcohol extract, water hood),while the lower part shows the protocol of extract preparation from plant seeds.

**Figure 2:**
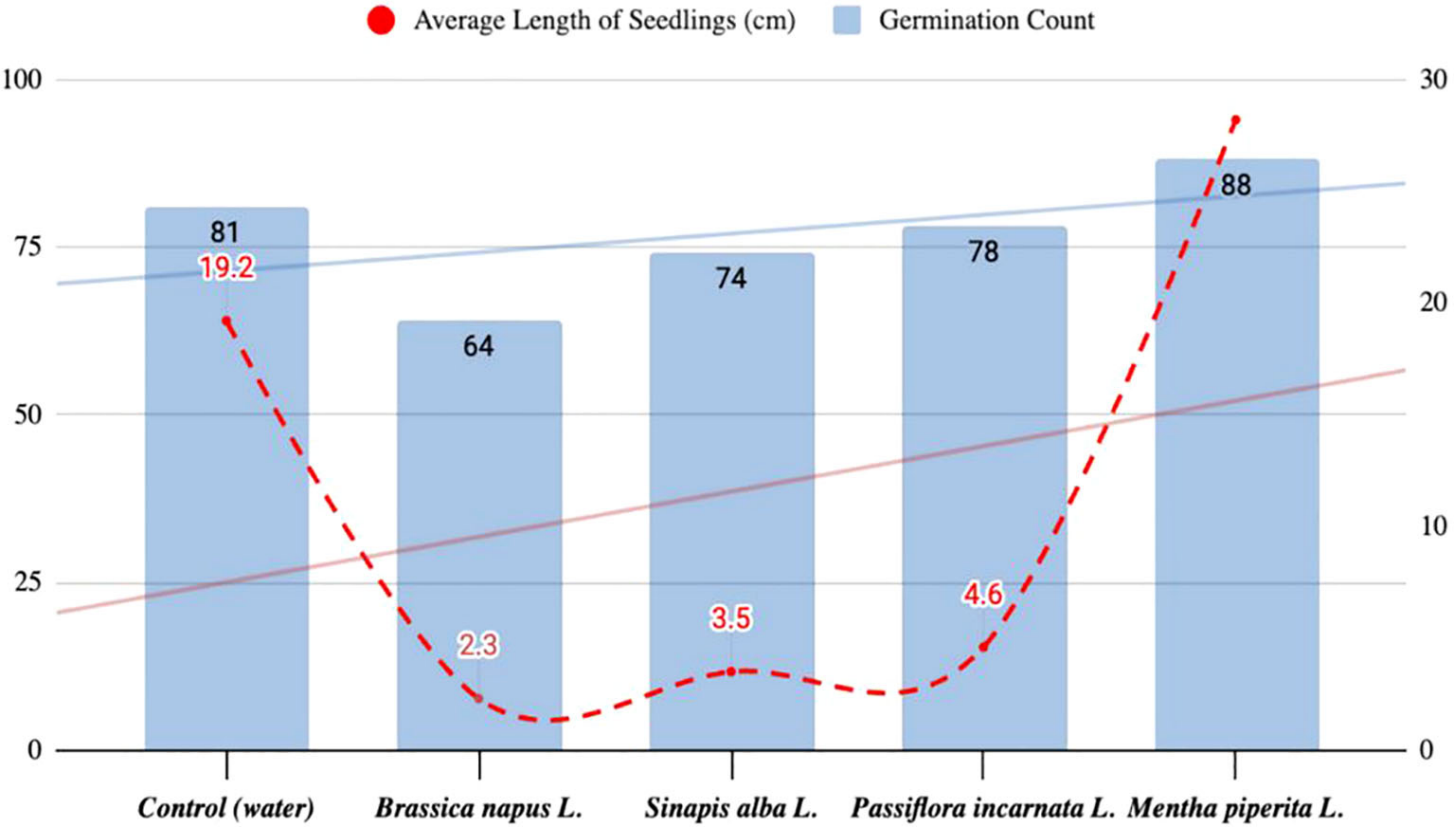
Results of the first model experiment of seed germination in Petri dishes.

The research methods included literature review, controlled experimental studies (see **Figure 1** for a diagram of the sequence of experiments) with a description of growth conditions, and mathematical and statistical data processing. The experiments were conducted over a period of 18 months. All experiments were performed in triplicate (n = 3). Data are presented as mean ± standard deviation (SD). One-way ANOVA was used to evaluate differences among treatment groups, followed by Tukey’s *post hoc* test for pairwise comparisons. A p-value < 0.05 was considered statistically significant. Statistical calculations were performed using standard statistical tools. In the laboratory, aqueous extracts were prepared by grinding 100 g of dried plant material (Brassica napus L., Mentha piperita L., Passiflora incarnata L., Sinapis alba L.), mixing it with 200 ml of hot distilled water (approx. 95 °C), and shaking on a laboratory shaker at 200 rpm for 15 minutes. This resulted in an approximate extract concentration of 500 mg/ml (dry weight basis). The obtained extract was filtered through cheesecloth, cooled, and used immediately for seed treatment. Tomato seeds were pre-selected for homogeneity and soaked in the respective aqueous extracts (prepared by mixing 100 g of dried plant material with 200 ml of hot distilled water, resulting in an approximate concentration of 500 mg/ml) for 2 hours at room temperature prior to germination assays. Petri dishes (Ø 90 mm) were filled with 5 ml of the test solutions, and the seeds were placed on filter paper along the diameter. The dishes were incubated in a thermostat at 26 ± 0.5 °C and 60% relative humidity. Germination was recorded after 24, 48, and 72 hours, and again on day 7. The experiment was repeated six times, with 100 seeds in each replicate. The average results are presented in **Figure 2**. Germination energy (GE) was calculated as the percentage of seeds that germinated within the first 24 hours relative to the total number of seeds germinated by day 7. This parameter indicates the speed and uniformity of germination and is commonly used in seed physiology studies (Šerá, 2023). A total of 88 seeds germinated in the group treated with *Mentha piperita L.* extract, with an average seedling length of 2.82 cm. In comparison, the control group showed 81 germinated seeds with an average length of 1.92 cm. Treatment with *Sinapis alba L.* resulted in 74 germinated seeds (3.5 cm), and *Brassica napus L.* resulted in 64 germinated seeds (2.3 cm). These results suggest that bioactive extracts have varying effects on tomato seed germination and early growth.

**Figure 3:**
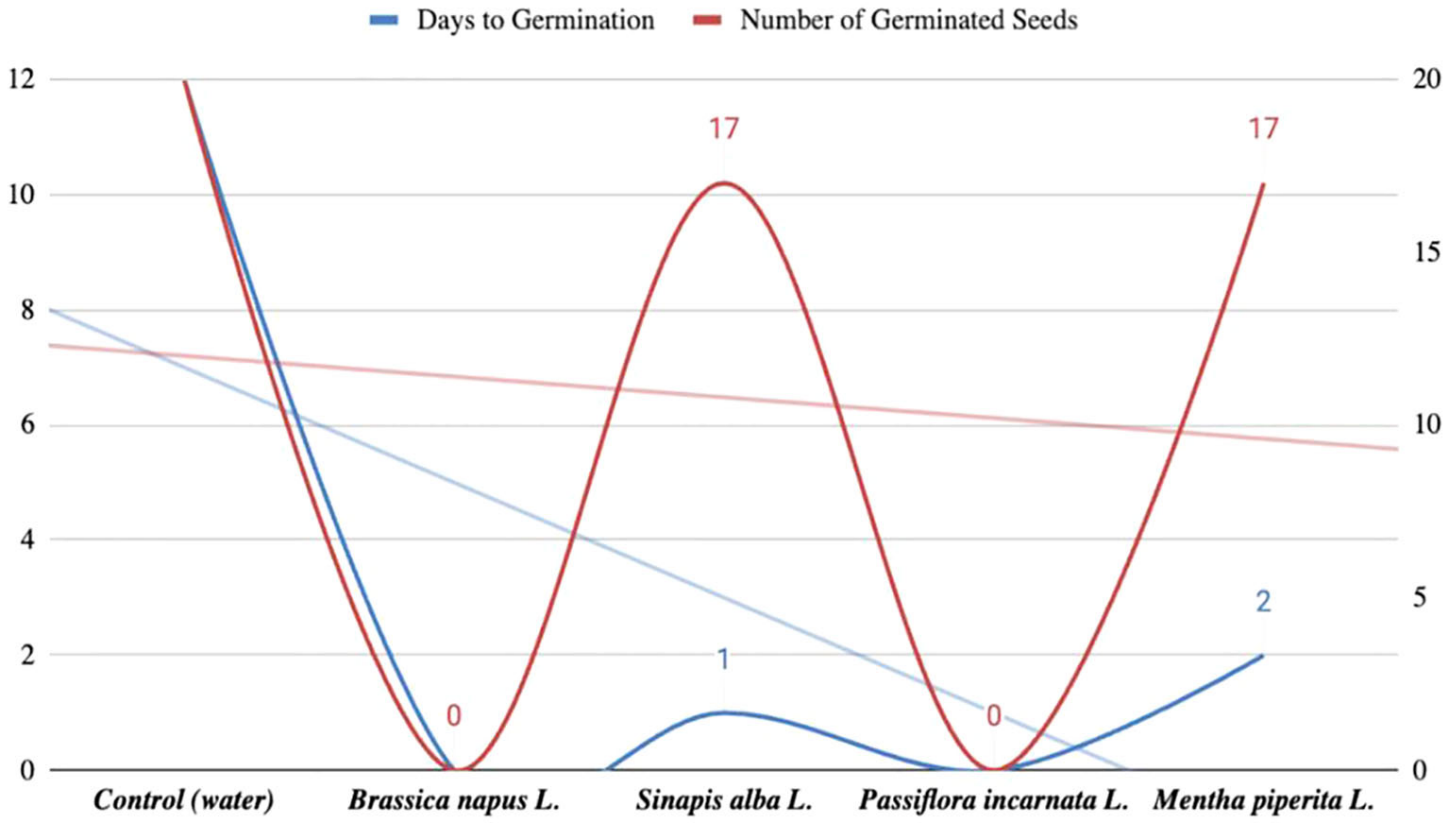
Results of the study of tomato seed germination in sand (12-day observation).

Based on these findings, we conducted an additional experiment using sand as the substrate to simulate more natural conditions. The seeds were again soaked in aqueous extracts, and germination was observed over 12 days. In the control group, seedlings appeared on day 12. In samples treated with *Mentha piperita L.* extract, 17 seeds germinated compared to 20 in the control. Samples treated with *Sinapis alba L.* showed germination one day later than the control. No seedlings appeared in samples treated with *Passiflora incarnata L.* or *Brassica napus L.* extracts. The results of this experiment are presented in **Figure 3**.

## 3 Analysis of the content of photosynthetic pigment in plants of *Solanum lycopersicum* L. treated with botanical extracts

**Figure 4:**
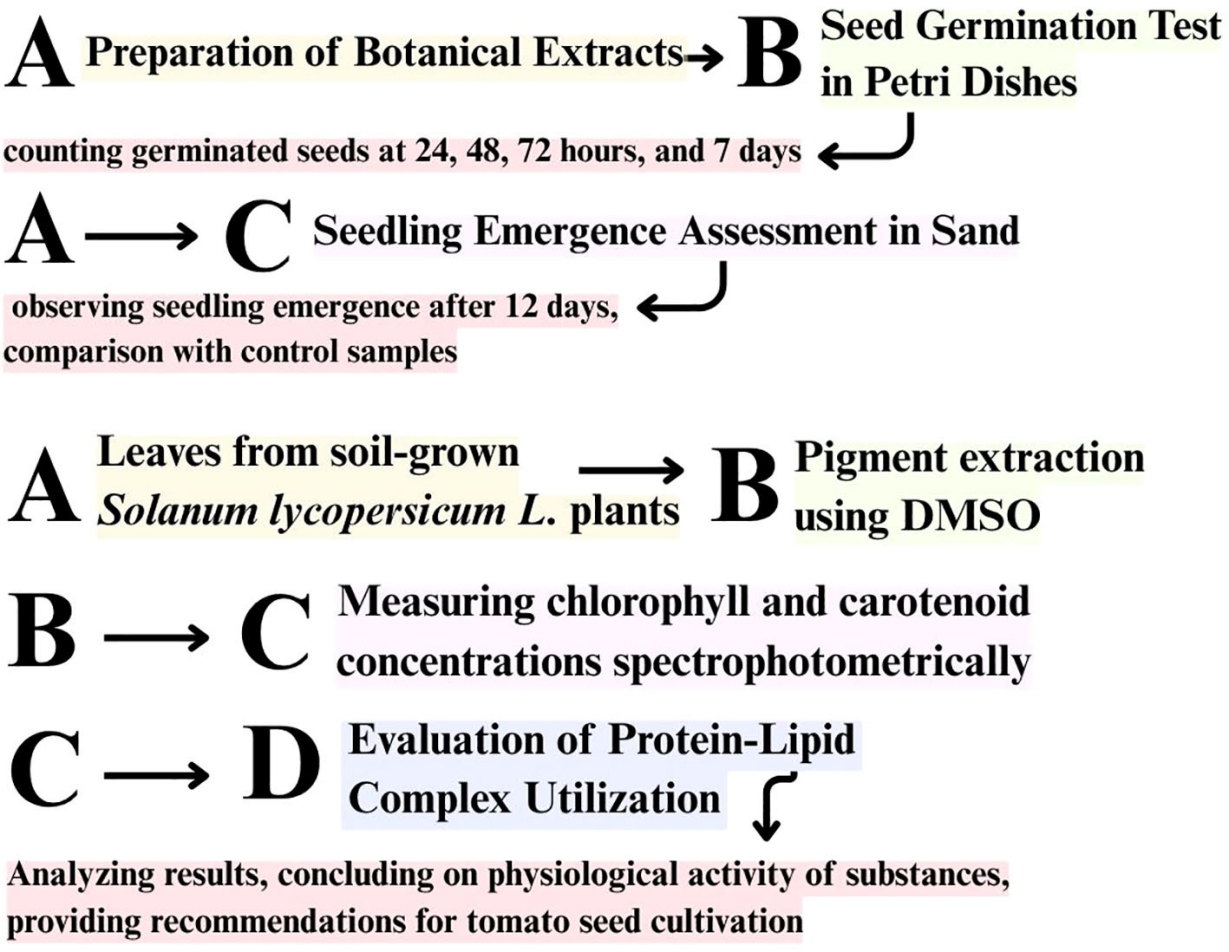
General experimental workflow illustrating the sequence of seed germination assays (in Petri dishes and sand) and photosynthetic pigment analysis using dimethyl sulfoxide (DMSO). The lower part of the scheme represents a separate pigment analysis performed on soil-grown tomato plants, independently of germination tests.

The content of photosynthetic pigments (chlorophyll a, chlorophyll b, total carotenoids) in plant material was determined by the spectrophotometric method of M.M. Musienko using the equipment ‘Spekol 11’ (Carl Zeiss/Jena, Germany). For laboratory studies, leaves of *Solanum lycopersicum* L. plants were selected from open ground in the flowering phase. Chlorophylls and carotenoids were extracted using dimethyl sulfoxide (DMSO) according to the method by Hiscox and Israelstam (1979), which allows efficient pigment extraction without maceration is presented in **Figure 4**. Although DMSO was used solely for pigment extraction and not as a treatment solvent, its potential effects were minimized by applying the same procedure across all samples.

This approach is commonly used in plant physiology studies due to its minimal pigment degradation. However, recent studies suggest that even low concentrations of DMSO can interfere with sulfur metabolism and plant biochemical responses, which should be considered when interpreting the results (Kaczor-Kamińska et al., 2024). It is important to note that this pigment analysis was conducted independently of the germination experiments. Tomato plants were grown under controlled conditions in open soil and treated with the aqueous extracts at the flowering stage. This approach allowed pigment measurements for all treatment variants, including *Passiflora incarnata* and *Brassica napus*, which had not produced seedlings during the sand-based germination trial. Photosynthetic pigments, including chlorophyll *a*, chlorophyll *b*, and carotenoids, were determined in plant extracts using dimethyl sulfoxide (DMSO) according to a modified in-house spectrophotometric method.

The optical density (A) of the extracts was measured at the following wavelengths corresponding to absorption peaks:


— chlorophylla: λ = 665 nm and λ = 649 nm

— carotenoids: λ = 480 nm

The concentrations of pigments (mg/g fresh weight) were calculated using the following formulas:


$$—\;Chlorophylla=\;12.19\;\times \;A_{665}\;-\;3.45\;\times \;A_{649}$$

$$—\;Chlorophyllb=\;21.99\;\times \;A_{649}\;-\;5.32\;\times \;A_{665}$$

$$—\;Carotenoids\;=\;(1000\;\times \;A_{480}\;-\;2.14\;\times \;Chl.a-\;70.16\;\times \;Chl.b)/220$$

According to the results of laboratory studies presented in **Table 1**, the highest chlorophyll *a* content was observed in tomato plants treated with seed extracts of *Sinapis alba* L. (6.73 ± 0.50 mg/g) and *Brassica napus* L. (4.93 ± 0.12 mg/g), while the lowest value was recorded in the control group (2.44 ± 0.09 mg/g). One-way ANOVA revealed statistically significant differences among treatments (*p* < 0.001). *Post hoc* analysis (Tukey’s test) confirmed that the increase in chlorophyll *a* content in all treated groups, especially in *Mentha piperita* L. and *Sinapis alba* L., was statistically significant compared to the control (see **Table 1**).

**Table 1:** Content of photosynthetic pigments (mg/g) in *Solanum lycopersicum* L. seedlings treated with botanical aqueous extracts.

Extract	Chlorophyll a (mg/g)	Chlorophyll b (mg/g)	Carotenoids (mg/g)
Control (untreated)	2,44 ± 0,09	1,53 ± 0,65	1,12 ± 0,08
*Brassica napus L.*	4,93 ± 0,12	4,10 ± 0,42	1,83 ± 0,22
*Sinapis alba L.*	6,73 ± 0,50**	6,21 ± 0,66**	1,89 ± 0,40*
*Passiflora incarnata L.*	5,12 ± 0,15*	4,98 ± 0,51*	1,75 ± 0,31*
*Mentha piperita L.*	5,80 ± 0,18**	5,21 ± 0,48**	2,00 ± 0,12*

Values are presented as mean ± SD (n = 3). Asterisks indicate significant differences compared to the control group based on one-way ANOVA with Tukey’s post hoc test (p < 0.05, p < 0.01).

The relative increase ranged from 34.5% to 45.9%. According to the results in **Table 2**, the most pronounced increase in chlorophyll *a* was observed in tomato plants treated with *Brassica napus L.* extract (+45.9%) and *Mentha piperita L.* extract (+38.6%). The highest concentration of chlorophyll *b* (6.21 ± 0.66 mg/g) was detected in plants treated with *Sinapis alba L.* extract, while the lowest (1.53 ± 0.65 mg/g) was recorded in the untreated control group (**Table 1**).

**Table 2:** Percentage increase in chlorophyll a content in tomato plants after treatment with botanical extracts.

Extract	Chlorophyll a increase (%)
Control (untreated)	-
*Brassica napus L.*	+45,9**
*Sinapis alba L.*	+43,7%**
*Passiflora incarnata L.*	+34,5%*
*Mentha piperita L.*	+38,6%**

Data represent percentage increase relative to control (n = 3). Asterisks indicate significant differences compared to the control group (one-way ANOVA with Tukey’s post hoc test: *p < 0.05, **p < 0.01).

Notably, a high chlorophyll *b* content is often associated with plant stress. Since flowering is a physiologically demanding phase, elevated chlorophyll *b* levels may reflect adaptive responses to stress. In contrast to chlorophylls, the carotenoid content showed a substantial increase, particularly in plants treated with *Mentha piperita L.* — by 78.6% (from 1.12 to 2.00 ± 0.12 mg/g). Similar trends were recorded in the *Sinapis alba L.* (+41.0%) and *Brassica napus L.* (+23.6%) groups (**Table 1**).

One-way ANOVA indicated significant differences between treatment groups (p < 0.05), and Tukey’s *post hoc* test confirmed pairwise significance. Asterisks in **Table 2** mark statistically significant differences compared to the control group.

The highest overall carotenoid level was found in *Mentha piperita L.*-treated plants (2.00 ± 0.12 mg/g), followed by *Sinapis alba L.* (1.89 ± 0.40 mg/g) and *Brassica napus L.* (1.83 ± 0.22 mg/g). These results indicate that depending on the extract, carotenoid accumulation may either increase or remain close to the control level, reflecting cultivar-specific physiological responses.

The maximum ratio of chlorophyll a/b, according to **Table 3**, was found in plants treated with seed extracts of *Brassica napus L.* (1.20) and *Mentha piperita L.* (1.42), while the minimum ratio was found in plants treated with *Passiflora incarnata L*. (1.03) and *Sinapis alba L*. (0.83). Regarding the ratio of total chlorophyll to carotenoid content — an important physiological marker reflecting the stress condition of plants — the treated variants showed an increase compared to the control. In particular, the highest values of this ratio were recorded in plants treated with *Sinapis alba* L. (6.85 vs. 3.54 in the control), *Passiflora incarnata* L. (5.77), *Mentha piperita* L. (5.51) and *Brassica napus* L. (4.93). One-way ANOVA revealed statistically significant differences in both the chlorophyll a/b and chlorophylls/carotenoids ratios (p < 0.05). Different letters in **Table 3** indicate significantly different group means according to Tukey’s *post hoc* test. The obtained results confirm that the treatment with seed extracts of *Brassica napus* L*., Mentha piperita* L*., Passiflora incarnata* L*. and Sinapis alba* L. contributes to the increase of photosynthetic pigments content in tomatoes.

**Table 3:** Ratio of chlorophyll a/b and chlorophylls to carotenoids in tomatoes after treatment with botanical extracts.

Extract	Chlorophyll a/b (± SD)	Chlorophylls/carotenoids (± SD)
Control (untreated)	1.60 ± 0.08a	3.54 ± 0.45a
*Brassica napus L.*	1.20 ± 0.05b	4.93 ± 0.29c
*Sinapis alba L.*	0.83 ± 0.04c	6.85 ± 0.50b
*Passiflora incarnata L.*	1.03 ± 0.06b	5.77 ± 0.45b
*Mentha piperita L.*	1.42 ± 0.09a	5.51 ± 0.38a

Values of chlorophylls to carotenoids ratio were recalculated based on total chlorophyll (a + b) divided by carotenoid content (mg/g), according to **Table 1**.

The high ratio of chlorophyll a to b indicates the potential role of these extracts in enhancing the effective photosynthetic activity of plants under stressful conditions. As for the change in vegetative mass growth, the best results were demonstrated by the extract of *Mentha piperita* L. By analyzing the germination energy of tomato seeds on the 7th day of the study, a significant difference between the control variant and the plants treated with the extracts was obtained.

## 4 Conclusions and future

The influence of botanical extracts led to an increase in the level of photosynthetic pigments in the leaves of *Solanum lycopersicum* L. under laboratory conditions. The highest content of chlorophyll a (6.73 ± 0.50 mg/g) and chlorophyll b (6.21 ± 0.66 mg/g) was observed in the *Sinapis alba* extract variant (p < 0.01), significantly exceeding the control values. Extracts of *Mentha piperita* and *Brassica napus* also significantly increased pigment concentrations. The percentage increase in chlorophyll a ranged from +34.5% to +45.9%, suggesting possible stimulation of chlorophyll biosynthesis.

Although *Passiflora incarnata* did not promote seedling emergence in the sand-based experiment, its aqueous extract contributed to an increase in photosynthetic pigment levels, indicating a potential physiological effect on pigment metabolism.

Aqueous extracts of *Brassica napus*, *Mentha piperita*, and *Sinapis alba* positively influenced seed germination and early seedling development under controlled laboratory conditions. Germination energy increased by 28–42% compared to the control, suggesting a possible role of these extracts as natural growth modulators.

One-way ANOVA and Tukey’s *post hoc* test confirmed statistically significant differences (p < 0.05) in pigment content, germination energy, and chlorophyll/carotenoid ratios between treatments. For instance, the lowest chlorophyll a/b ratio (0.83) was recorded in the *Sinapis alba* variant, suggesting increased chlorophyll b participation. The highest total chlorophyll to carotenoids ratio was observed in plants treated with *Sinapis alba* (6.85), followed by *Passiflora incarnata* (5.77) and *Mentha piperita* (5.51).

These findings provide preliminary evidence that seed-derived botanical extracts can affect early physiological responses and photosynthetic pigment accumulation in tomatoes under *in vitro* conditions. However, further studies are necessary to validate these results under field conditions, including assessments of biomass production, stress biomarkers, and yield outcomes before broad recommendations can be made for agricultural practice.

## Data Availability

The original contributions presented in the study are included in the article/supplementary material. Further inquiries can be directed to the corresponding authors.

## References

[B1] Ahmed S. A. El-Dabaa M. A. T. Messiha N. K. El-Masry R. R. Dawood M. G. (2024). The allelopathic influence of the seed powder of Brassica napus (canola) in controlling Orobanche crenata (broomrape) infesting Pisum sativum (pea) plants. J. Materials Environ. Sci. 15, 464–475.

[B2] Chalkos D. Kadoglidou K. Karamanoli K. Fotiou C. Pavlatou-Ve A. Eleftherohorinos I. . (2010). Mentha spicata and Salvia fruticosa composts as soil amendments in tomato cultivation. Plant Soil 332, 495–509. doi: 10.1007/s11104-010-0315-4

[B3] Chou C.-H. Wang C.-M. (2023). Allelopathy research: Past, present and future I. Allelopathy in natural ecosystems. Allelopathy J. 58, 1. doi: 10.26651/allelo.j/2023-58-1-1416

[B4] Grodzinsky A. M. (1997). Fundamentals of Plant Chemical Interaction (Kyiv: Naukova Dumka).

[B5] Hierro J. L. Callaway R. M. (2021). The ecological importance of allelopathy, Annual Review of Ecology. Evolution Systematics 52, 25–45. doi: 10.1146/annurev-ecolsys-051120-03061

[B6] Hussain M. I. Danish S. Sánchez-Moreiras A. M. Vicente Ó. Jabran K. Chaudhry U. K. . (2021). Unraveling Sorghum allelopathy in agriculture: Concepts and implications. Plants 10, 1795. doi: 10.3390/plants10091795, PMID: 34579328 PMC8470078

[B7] Jabran K. (2017). “Brassicaceae allelopathy for weed control,” in Manipulation of Allelopathic Crops for Weed Control (Springer, Cham). doi: 10.1007/978-3-319-53186-1_3

[B8] Kaczor-Kamińska M. Kaszuba K. Bilska-Wilkosz A. Iciek M. Wróbel M. Kamiński K. (2024). Dimethyl sulfoxide (DMSO) as a potential source of interference in research related to sulfur metabolism—A preliminary study. Antioxidants 13, 582. doi: 10.3390/antiox13050582, PMID: 38790687 PMC11117631

[B9] Kalisz S. Kivlin S. N. Bialic-Murphy L. (2021). Allelopathy is pervasive in invasive plants. Biol. Invasions 23, 367–371. doi: 10.1007/s10530-020-02448-7

[B10] Khamare Y. Khamare Y. Chen J. Marble S. C. (2022). Allelopathy and its application as a weed management tool: A review. Front. Plant Sci. 13. doi: 10.3389/fpls.2022.1034649, PMID: 36518508 PMC9742440

[B11] Khanh T. D. Hong N. H. Xuan T. D. Chung I. M. (2005). Paddy weed control by medicinal and leguminous plants from Southeast Asia. Crop Prot. 24, 421–431. doi: 10.1016/j.cropro.2004.09.020

[B12] Kostina-Bednarz M. Płonka J. Barchanska H. (2023). Allelopathy as a source of bioherbicides: challenges and prospects for sustainable agriculture. Rev. Environ. Sci. Bio/Technology 22, 471–504. doi: 10.1007/s11157-023-09617-3

[B13] Krupa L. I. Figurskaya A. A. (1990). “Phenolic compounds in soil under cereal crops,” in Allelopathy and Plant Productivity (Naukova Dumka, Kyiv), 46–50.

[B14] Latif S. Chiapusio G. Weston L. A. (Eds.) (2017). “Allelopathy and the role of allelochemicals in plant defense,” in Advances in Botanical Research, vol. 82 (Amsterdam, Netherlands: Elsevier), 19–54. doi: 10.1016/bs.abr.2016.12.001

[B15] Li Z. R. Amist N. Bai L. Y. (2019). Allelopathy in sustainable weed management. Allelopathy J. 48, 109–138. doi: 10.26651/allelo.j/2019-48-2-1249

[B16] Liu S. Wu F. Wen X. (2013). Allelopathic effects of root exudates of Chinese onion on tomato growth and the pathogen *Fusarium oxysporum* (Sch1) f.sp. *lycopersici* . Allelopathy J. 31, 387–403.

[B17] Šerá B. (2023). Methodological contribution on seed germination and seedling initial growth tests in wild plants. Notulae Botanicae Horti Agrobotanici Cluj-Napoca 51, 13164. doi: 10.15835/nbha51213164

[B18] Shirgapure K. H. Ghosh P. (2020). Allelopathy: A tool for sustainable weed management. Arch. Curr. Res. Int. 20, 17–25. doi: 10.9734/acri/2020/v20i330180

[B19] Xu Y. Chen X. Ding L. Kong C.-H. (2023). Allelopathy and allelochemicals in grasslands and forests. Forests 14, 562. doi: 10.3390/f14030562

[B20] Zhang Z. Liu Y. Yuan L. Weber E. van Kleunen M. (2020). Effect of allelopathy on plant performance: A meta-analysis. Ecol. Lett. 24, 348–362. doi: 10.1111/ele.13627, PMID: 33085152

