# Supplementary figures and images for: Allelopathic effects of bioactive aqueous extracts on the growth and development of Solanum lycopersicum L
Source: Front Plant Sci. 2025 Jul 28;16:1536309. doi: 10.3389/fpls.2025.1536309 (PMC12336125; doi:10.3389/fpls.2025.1536309)

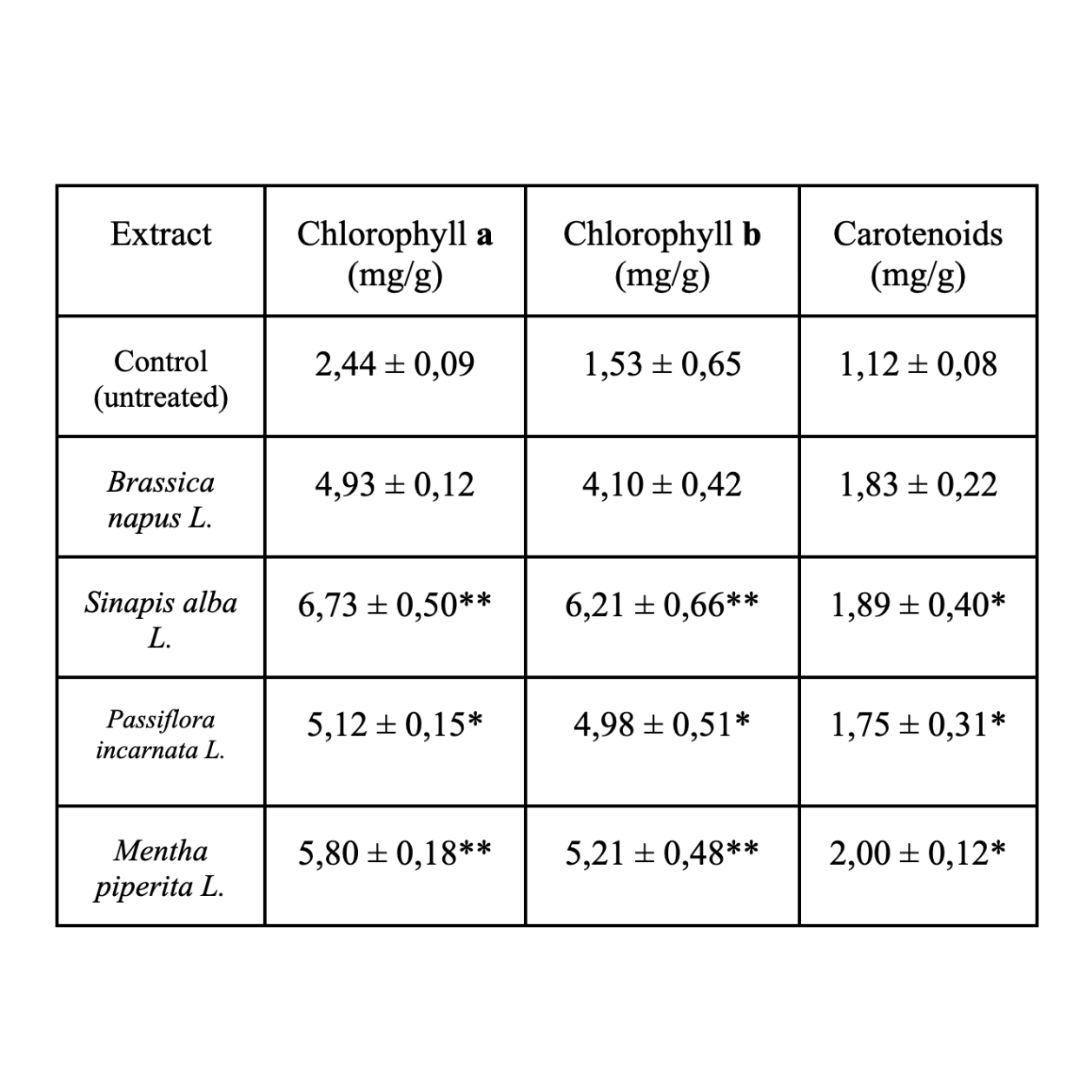

Supplement: Supplementary file 1 [file Image1.jpeg]

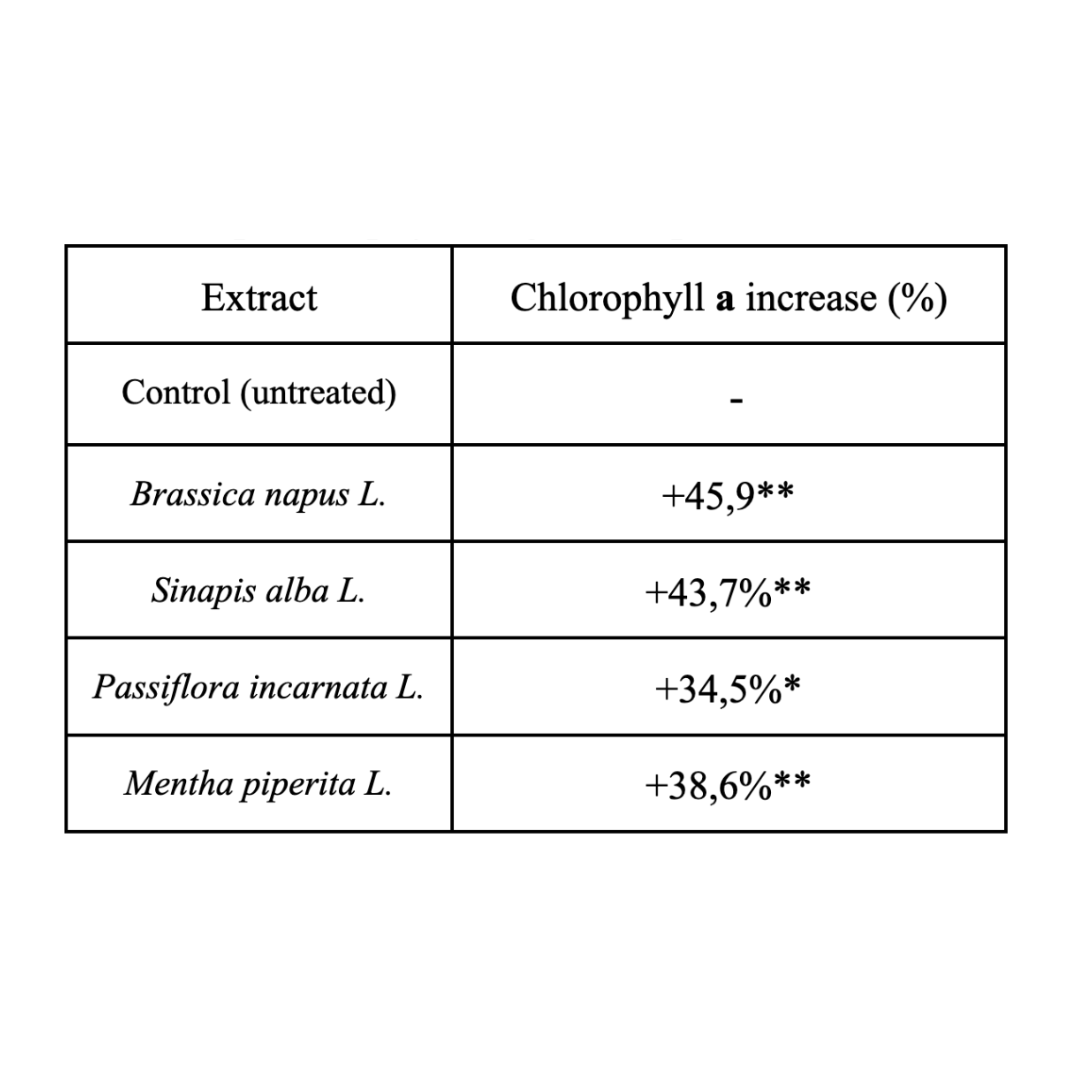

Supplement: Supplementary file 2 [file Image2.png]

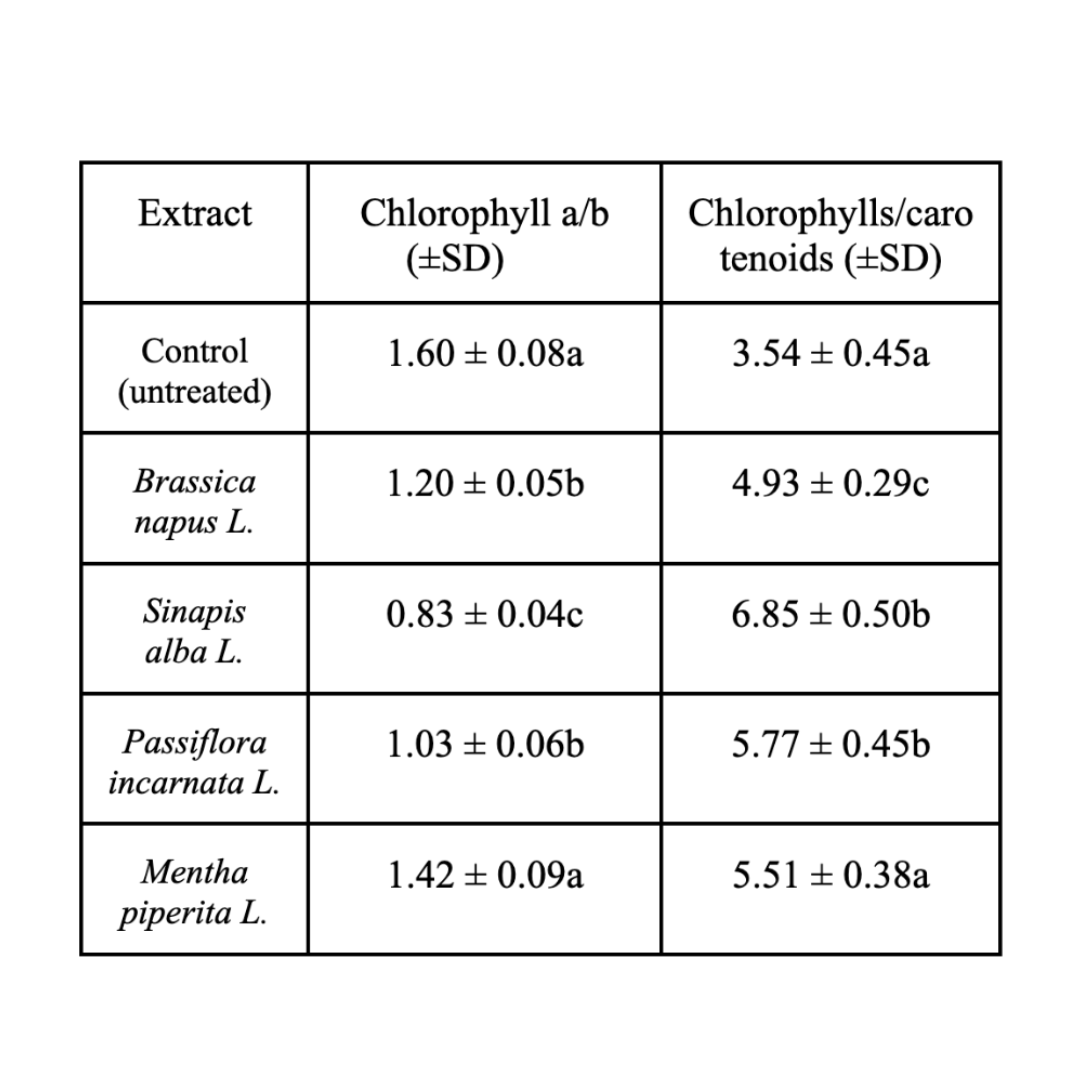

Supplement: Supplementary file 3 [file Image3.png]
